# Supplementary material for: Aptamer Targets Triple-Negative Breast Cancer through Specific Binding to Surface CD49c
Source: Cancers (Basel). 2022 Mar 18;14(6):1570. doi: 10.3390/cancers14061570 (PMC8946172; doi:10.3390/cancers14061570)
Supplement: Supplementary file 1 [file cancers-14-01570-s001.zip › cancers-1593749- Supplementary material for xml.pdf]

Supplemental Material and Methods

Reagents and Chemicals

All reagents were ACS grade or higher. All solvents used, including water, were LC/MS grade. Ammonium bicarbonate (ABC), trifluoroacetic acid (TFA), potassium ferricyanide, sodium thiosulfate, and acetic acid were purchased from Sigma-Aldrich, St. Louis, MO, USA. Iodoacetamide (IDA), dithiothreitol (DTT), acetonitrile (ACN), formic acid, and methanol were purchased from Thermo Scientific, Waltham, MA, USA. Sequencing-grade modified trypsin (Promega, Madison, WI, USA) was used.

In-gel Digestion and Protein Identification

The proteins in the gel were digested as described previously with slight modification [16]. After SDS-PAGE, the gel was stained with a silver staining kit (Pierce™ Silver Stain kit; Thermo Scientific, Waltham, MA). The target bands were excised from the gel and destained with 30 mM potassium ferricyanide and 100 mM sodium thiosulfate (Sigma-Aldrich). The gel bands were then suspended in 200 μL of 10 mM DTT for 30 min followed by 200 μL of IDA for 60 min. The gel bands were washed with 25 mM ABC-50% MeOH once, with 10% acetic acid-50% MeOH three times, and then with water once. The gels were dehydrated with acetonitrile and dried in a SpeedVac (Thermo Scientific). Then, 200 μL of 20 mM ABC and an 8-μg aliquot of trypsin in 50 mM ABC (pH 8.0) were added to cover the gel pieces before incubation overnight at 37 °C. After trypsin digestion, the supernatant was collected, and the peptides were extracted from the gels with 50% ACN-5% TFA and 75% ACN-0.1% TFA. All the supernatants were combined and dried in a SpeedVac.

LC-MS/MS Analysis

A nanoflow ultra-high performance liquid chromatography instrument (Easy-nLC, Thermo Scientific) was coupled on-line to a Q Exactive mass spectrometer (Thermo Scientific) with a nanoelectrospray ion source (Thermo Scientific). Peptides were loaded onto a C18 reversed-phase column (25 cm long, 75 μm inner diameter) and separated with a linear gradient of 5–35% buffer B (100% acetonitrile in 0.1% formic acid) at a flow rate of 300 nL/min over 60 min. Each sample was analyzed by LC-MS/MS three times. MS data were acquired using a data-dependent top15 method, which dynamically chose the most abundant precursor ions from the survey scan (350–1600 m/z) using HCD fragmentation. Survey scans were acquired at a resolution of 70,000 at m/z 400. Unassigned precursor ion charge states, as well as singly charged species, were excluded from fragmentation. The isolation window was set to 3 Da and fragmented with normalized collision energies of 28. The maximum ion injection times for the survey scan and the MS/MS scans were 20 ms and 120 ms, respectively, and the ion target values were set to 1E6 and 1E5, respectively. Selected sequenced ions were dynamically excluded for 30 seconds. Data were acquired using Xcalibur software (Thermo Scientific).

Data Processing and Bioinformatic Analysis

Mass spectrometry raw data were analyzed using MaxQuant software version 1.5.2.8 and the Andromeda search engine [17,18]. The initial maximum allowed mass deviation was set to 10 ppm for monoisotopic precursor ions and 0.5 Da for MS/MS peaks. Enzyme specificity was set to trypsin, defined as C-terminal to arginine and lysine excluding proline, and a maximum of two missed cleavages was allowed. Carbamidomethyl cysteine was set as a fixed modification. N-terminal acetylation and methionine oxidation were set as variable modifications. The spectra were searched by the Andromeda search engine against the human sequence database (containing 20,193 human protein entries) combined with 248 common contaminants and concatenated with the reversed versions of all sequences. Protein identification required at least one unique or razor peptide per protein group. Quantification in MaxQuant was performed using the built-in XIC-based label-free quantification (LFQ) algorithm [18]. The required false positive rate for identification was

set to 1% at the peptide and 1% at the protein level, and the minimum required peptide length was set to six amino acids. Contaminants, reverse identification, and proteins only identified by site were excluded from further data analysis. The LFQ values were log_2-transformed. After filtering (at least three valid LFQ values in at least one group), the remaining missing LFQ values were imputed from a normal distribution (width: 0.3; downshift: 1.8). Student's two-sample t-test was used to assess the statistical significance of protein abundances using 1% permutation-based FDR adjustment [19].

Table S1. Identification of differential proteins between the PDGC21T aptamer and random ssDNA coprecipitates using LC-MS/MS analysis.

(attached "Table S1.xlsx" file).

Table S2. Quantification analysis for the differential proteins.

(attached "Table S2.xls" file).

Table S3. Figure 5 related median fluorescence intensity data.

	**Anti-CD49c**	**Aptamer**	**IgG**
MDA-MB-157	839	218	25.6
SUM159PT	268	185	16.7
HCC1937	611	442	23.8
MDA-MB-231	277	360	15.0
HCC38	959	432	25.5
MDA-MB-436	221	216	16.7
MDA-MB468	81,7	75.6	23.6
BT549	236	360	58.9
Hs578T	134	158	22.0
HCC70	215	238	16.7
T47D	44	122	45.7
MCF7	29.9	50.1	9.67
AsPC-1	122	150	95
MDA-Panc-28	410	136	551
H9	50.1	15.8	13.2
Jeko-1	15.8	13.2	16.7
Jurkat	7.05	6.16	7.91
Mino	7.05	35.2	29.0

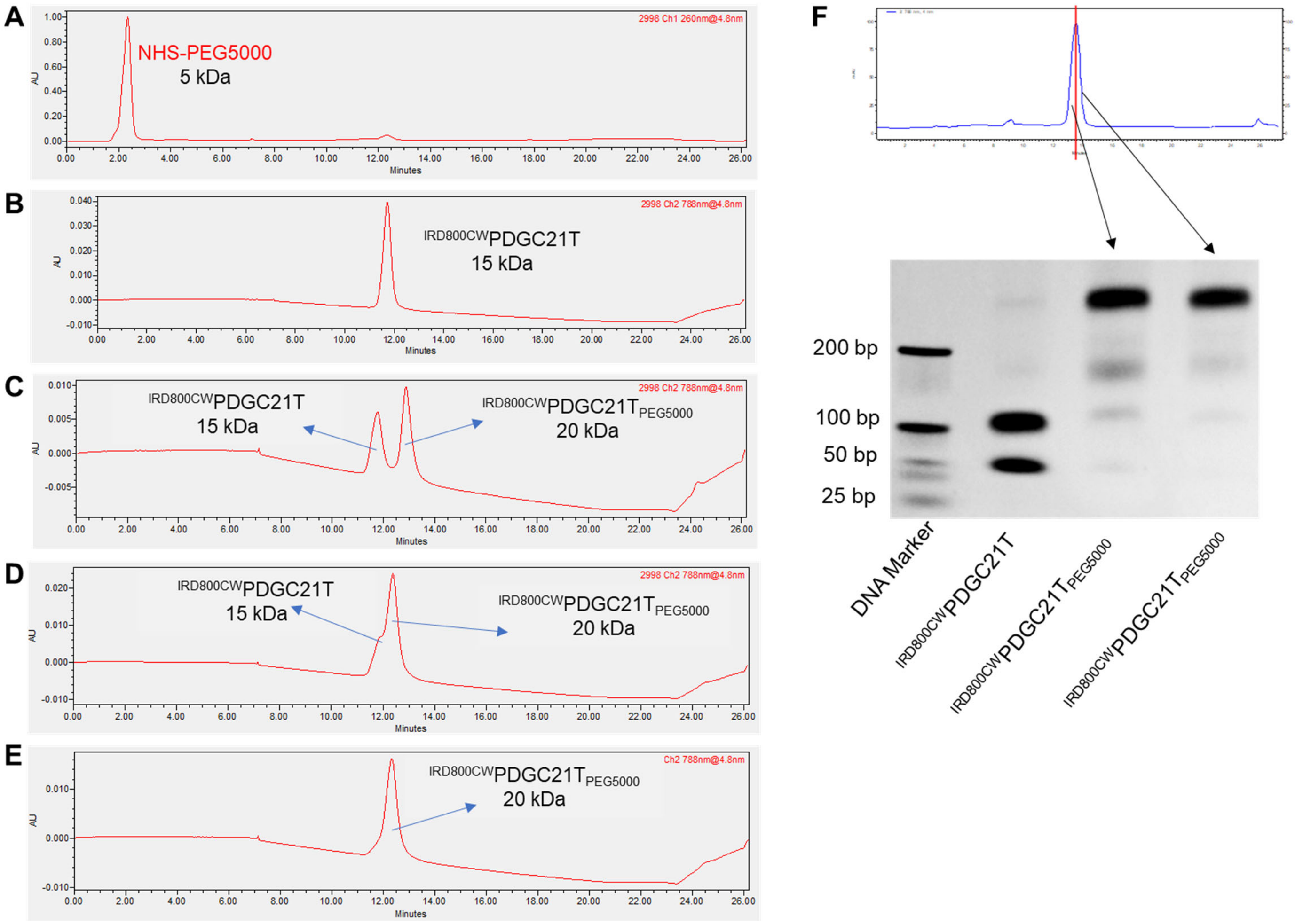

Figure S1. PEGylation process for PDGC21T aptamer. (**A, B**) HPLC for NHS-PEG5000 and ^{IRD800CW}Aptamer. (**C–E**) Different molar ratios of NHS-PEG5000 to IRDy800CW yield different productions. Ratios shown are 50:1 (**C**), 240:1 (**D**), and 480:1 (**E**). (**F**) Purified ^{IRD800CW}Aptamer$_{PEG5000}$ identification using SDS-PAGE electrophoresis.

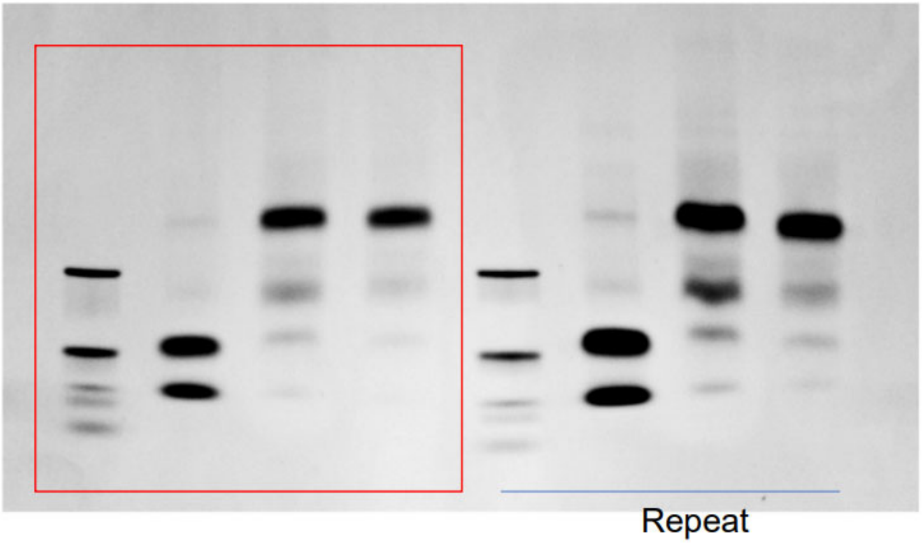

Figure S2: Uncropped Western blot image from Figure S1F. Red box indicates the area shown in Figure S1F.

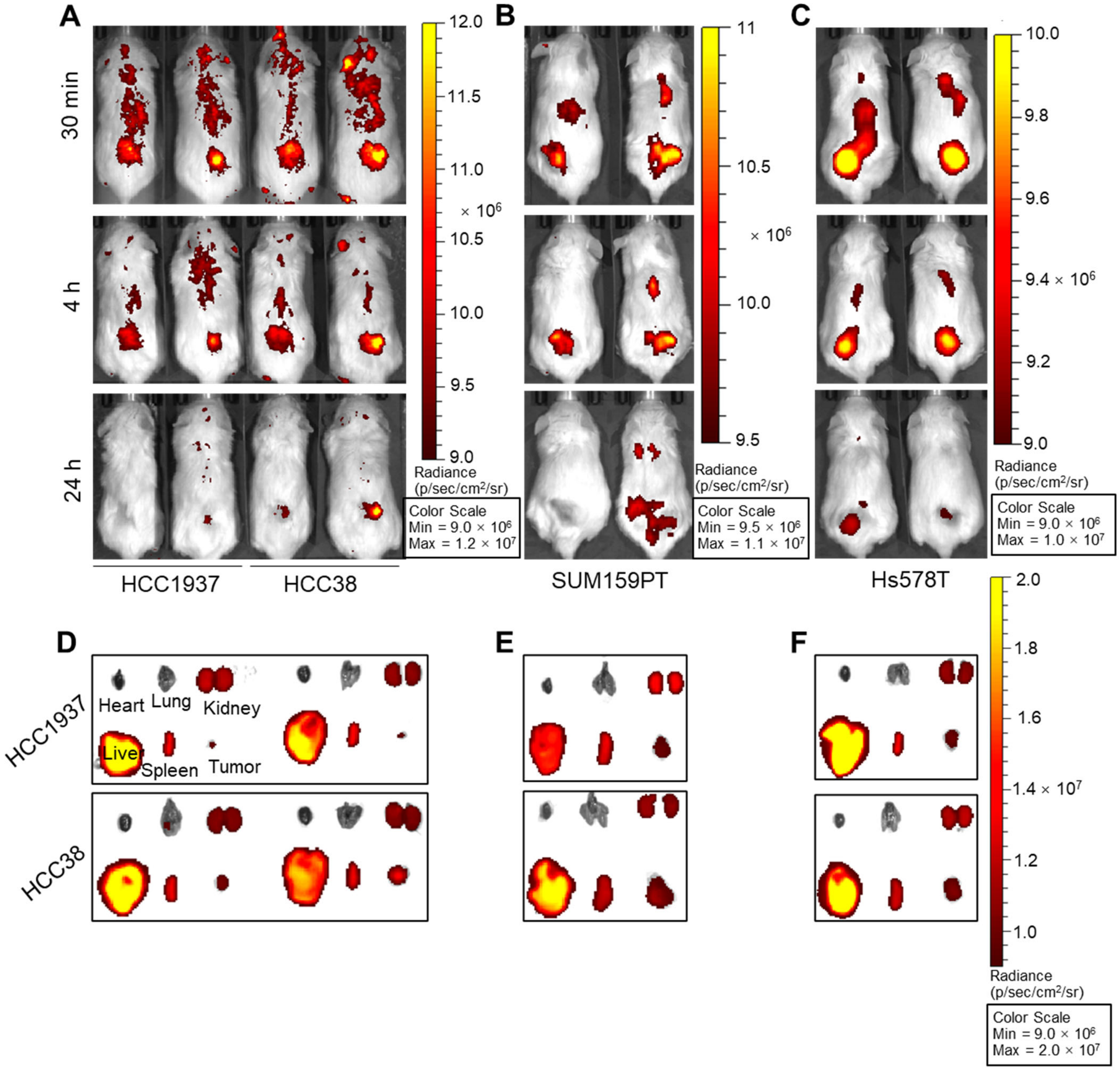

Figure S3. The PEGylated PDGC21T aptamer targeted and remained within HCC1937, HCC38, SUM159PT, and Hs578T xenograft tumors. Cells were implanted into the lower back of NSG mice. After the tumor volume reached 0.5 cm^3, 66.7 pmol/mouse of ^{IRD800CW}Aptamer$_{PEG5000}$ was intravenously administered to tumor-bearing mice. At 30 min, 4 hours, and 24 hours post-injection, mice were imaged in vivo. (**A–C**) The ^{IRD800CW}Aptamer$_{PEG5000}$ targeted HCC1937 (**A**), HCC38 (**A**), SUM159PT (**B**), and Hs578T (**C**) xenograft tumors. (**D–F**) Ex vivo imaging for hearts, lungs, kidneys, livers, spleens, and tumors of ^{IRD800CW}Aptamer$_{PEG5000}$ injected HCC1937 (**D**), HCC38 (**D**), SUM159PT (**E**), and Hs578T (**F**) tumor-bearing mice. Mice were euthanized 24 hours post-aptamer injection.

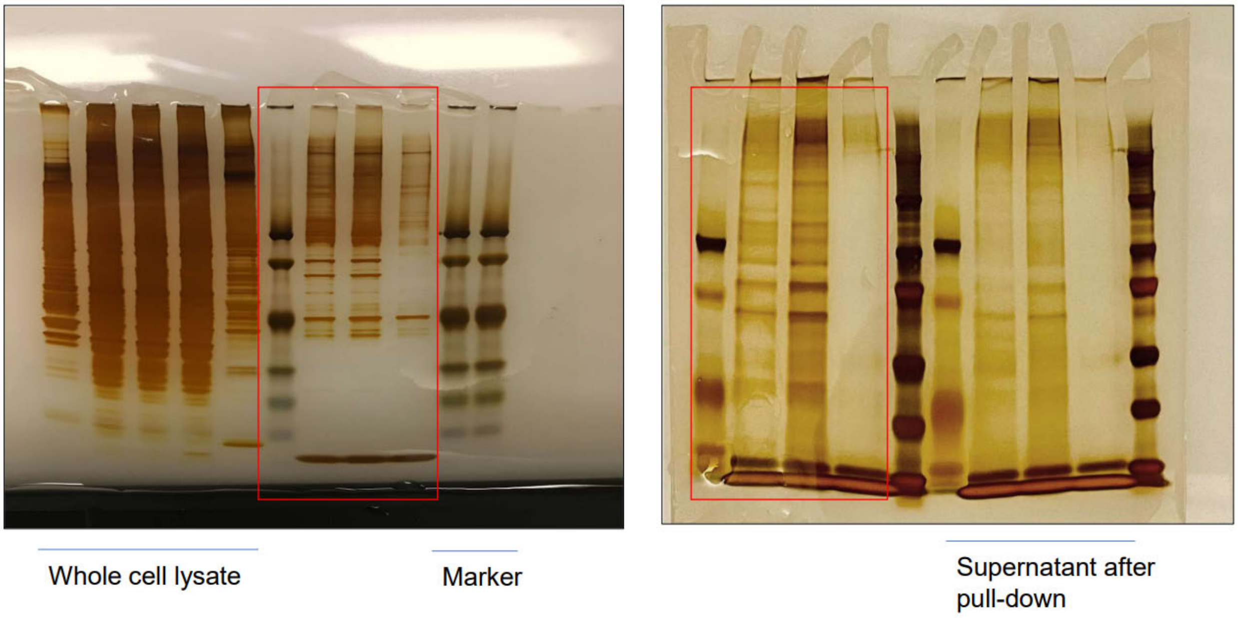

Figure S4. Uncropped Western blot from Figure 4B. Red boxes indicate the areas shown in Figure 4B.

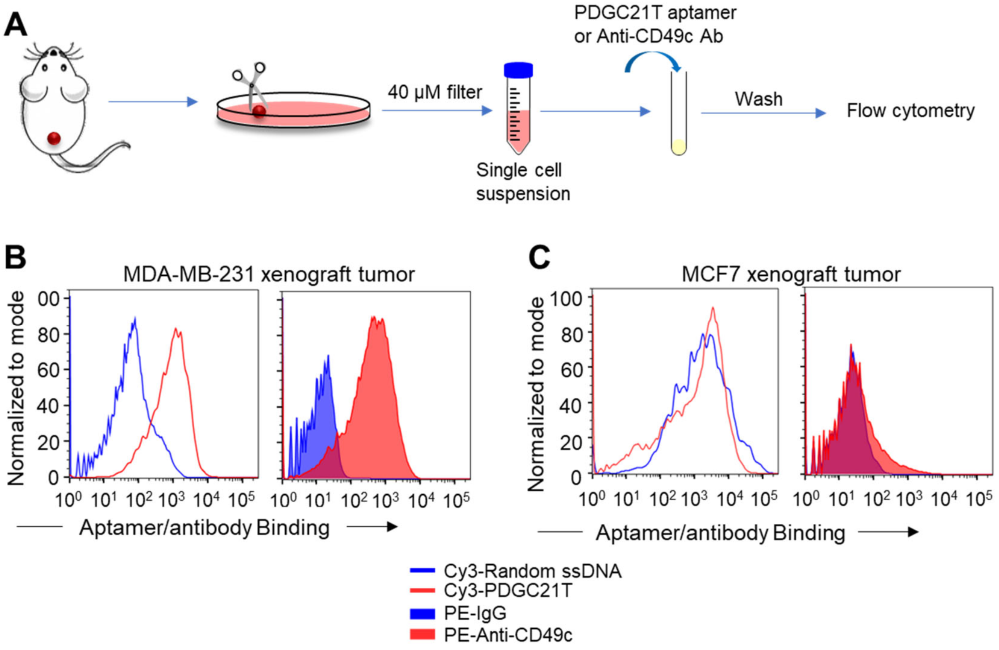

Figure S5. Ex vivo binding test of the PDGC21T aptamer and anti-CD49c to xenograft-tumor cells. (**A**) Schematic depicting the procedures of tumor cell isolation and the binding test for the PDGC21T aptamer and anti-CD49c. (**B, C**) Flow cytometry test for the binding of the PDGC21T aptamer and anti-CD49c to MDA-MB-231 (TNBC) (**B**) and MCF7 (non-TNBC) (**C**) xenograft tumor cells.

References

16. Zhang, Y.; Sun, H.; Zhang, J.; Brasier, A.R.; Zhao, Y. Quantitative Assessment of the Effects of Trypsin Digestion Methods on Affinity Purification–Mass Spectrometry-based Protein–Protein Interaction Analysis. *J. Proteome Res.* **2017**, *16*, 3068–3082.
17. Cox, J.; Mann, M. MaxQuant enables high peptide identification rates, individualized p.p.b.-range mass accuracies and proteome-wide protein quantification. *Nat. Biotechnol.* **2008**, *26*, 1367–1372.
18. Cox, J.; Hein, M.Y.; Luber, C.A.; Paron, I.; Nagaraj, N.; Mann, M. Accurate proteome-wide label-free quantification by delayed normalization and maximal peptide ratio extraction, termed MaxLFQ. *Mol. Cell. Proteom.* **2014**, *13*, 2513–2526.

19. Tusher, V.G.; Tibshirani, R.; Chu, G. Significance analysis of microarrays applied to the ionizing radiation response. *Proc. Natl. Acad. Sci. USA* **2001**, *98*, 5116–5121.
